# A “No-Cure, No-Pay” Decision

**Published:** 1884-12

**Authors:** 


A "NO-CURE, NO-PAY" DECISION.

We clip the following from the Washington *Star:*

"A rather unusual case was submitted to Judge Hagner, in the Circuit Court, to-day. This was a suit to recover from Dr. W. H. Hale $100 paid him by a colored man named Meredith, under a guarantee of Hale that for that sum he would cure Meredith's son of pulmonary consumption, and in case he failed he would refund the money. The boy died, and Meredith sued Hale before a justice of the peace and obtained judgment. The case was reopened and judgment was given against Hale a second time, and he appealed. After the testimony ot the parties was given this morning, Judge Hagner affirmed the judgment below. He declined to consider the defendant's bill in bar for attending Meredith's wife, and decided the case solely as to the original claim. Judge Hagner read the heading ot Dr. Hale's circular—'Hearth and Home, sworn circulation 63,000 copies monthly'—and the signed guarantee to cure the son, and said this guarantee was to perform an impossibility—to reverse a decree of the Almighty—and was akin to gypsying and voudooism, which says: 'I dont't want your money, but as I must touch silver I must have it before I can succeed.' The justice was right in his decision that the money should be paid back, and this court affirms that decision."

The common law of contracts, applied in this case, resulting in the discomfiture of an advertising quack, reaffirms the status of the medical contract. If a physician agrees to cure a patient, and a consideration passes, the transaction becomes a contract, and the physician failing to perform his part, an action for recovery will lie.

